# Engineering and monitoring cellular barrier models

**DOI:** 10.1186/s13036-018-0108-5

**Published:** 2018-09-12

**Authors:** Jose Yeste, Xavi Illa, Mar Alvarez, Rosa Villa

**Affiliations:** 1 Institut de Microelectrònica de Barcelona, IMB-CNM (CSIC), 08193, Bellaterra, Barcelona, Spain; 2 CIBER de Bioingeniería, Biomateriales y Nanomedicina (CIBER-BBN), Barcelona, Spain

**Keywords:** Biological barriers, Cell barrier function, Microphysiological systems, Transepithelial electrical properties

## Abstract

Epithelia and endothelia delineate tissue compartments and control their environments by regulating the passage of ions and solutes. This barrier function is essential for the development and maintenance of multicellular organisms, and its dysfunction is associated with numerous human diseases. Recent advances in biomaterials and microfabrication technologies have evolved in vitro approaches for modelling biological barriers. Current microphysiological systems have become more efficient and reliable in mimicking the cell microenvironment. Additionally, methods for the quantification of barrier permeability have long provided significant insight into their underlying mechanisms. In this review, we outline the current techniques to quantify the barrier function of engineered tissues, and we also give an overview of recent microphysiological systems of biological barriers that emulate the microenvironment and microarchitecture of native tissues.

## Background

Physical barriers to separate different compartments are essential for the development and maintenance of multicellular organisms and are integral to numerous organs. Epithelia and endothelia form these vital barriers. They delineate tissue compartments and control their environments by regulating the passage of ions and solutes [1]. Some examples are renal tubule epithelium and blood capillaries. The physiological function of these biological barriers is diverse among tissues and responds to the particular needs of each organ, including the supply of nutrients, the absorption of ions, the secretion of waste, the protection against toxins, and the filtration of fluids. The deregulation of their essential function can lead to serious health complications. Numerous endothelia and epithelia dysfunctions are associated with prevalent human diseases such as hereditary diseases (e.g., hypomagnesemia), gastrointestinal tract diseases (e.g., Crohn’s disease), or viral infections (e.g., hepatitis C) [2–4].

Recent advances in microtechnologies and biomaterials have provided a new set of tools to construct relevant microphysiological systems (MPS) [5, 6]. These engineered devices aim to recapitulate tissues- and organ-level functions and are promising constructs for disease modelling and drug development applications. Although cell cultures may not capture all of the complexity of the in vivo system, the barrier models being developed are enhancing our ability to more closely mimic the in vivo environment and therefore better predict cell behaviour in vivo. To date, this technology has led to the engineering of diverse systems for modelling human diseases [7] and also for mimicking biological barriers of the lung [8], kidney [9], and brain [10], among others. Since the major function of a cell barrier is to regulate and to separate two distinct physiological compartments, the strategy to build more relevant in vitro models usually lies in compartmentalization of different environments [11]. Most engineered cell barrier approaches utilize physical interfaces for such propose and to support cells (e.g., permeable membranes or gel-liquid interfaces).

Besides compartmentalization, quantifying the permeability of barrier tissues is necessary to assess the state of the barriers and identify the factors contributing to barrier dysfunction. For example, in toxicology, the monitoring of the barrier integrity permits to evaluate the effects of toxic compounds; in disease modelling, to examine a barrier breakdown during a disease progression; or in drug development, to test the ability of new drugs to cross the barriers. In addition to tracer assays and immunocytochemistry, transepithelial electrical measurements—performed with extracellular electrodes in apical and basal sides—have been an essential methodology to quantify ion permeability and to elucidate important epithelial properties. These electrical measurements are non-invasive and yield information about the voltage, resistance, and current across the epithelium. Measuring these parameters under special conditions, it is possible to determine ion transepithelial transports and the electromotive forces generated by active transporters. Transepithelial electrical properties have also been widely used to determine ion selectivity, ion permeability, and electrophysiological characterization of epithelial tissues [12–14].

In this review, after introducing how epithelial and endothelial cells form functional barriers and describing some vital biological barriers of the human body, we outline the current techniques to quantify the barrier function of engineered tissues, focusing on permeability assays and transepithelial electrical measurements. Then, we give and overview of recent MPS for modelling biological barriers that emulate the cell microenvironment and microarchitecture of native tissues. Finally, we discuss the future perspectives in engineering and monitoring epithelial and endothelial barrier models.

## Epithelial and endothelial tissues

**Fig. 1:**
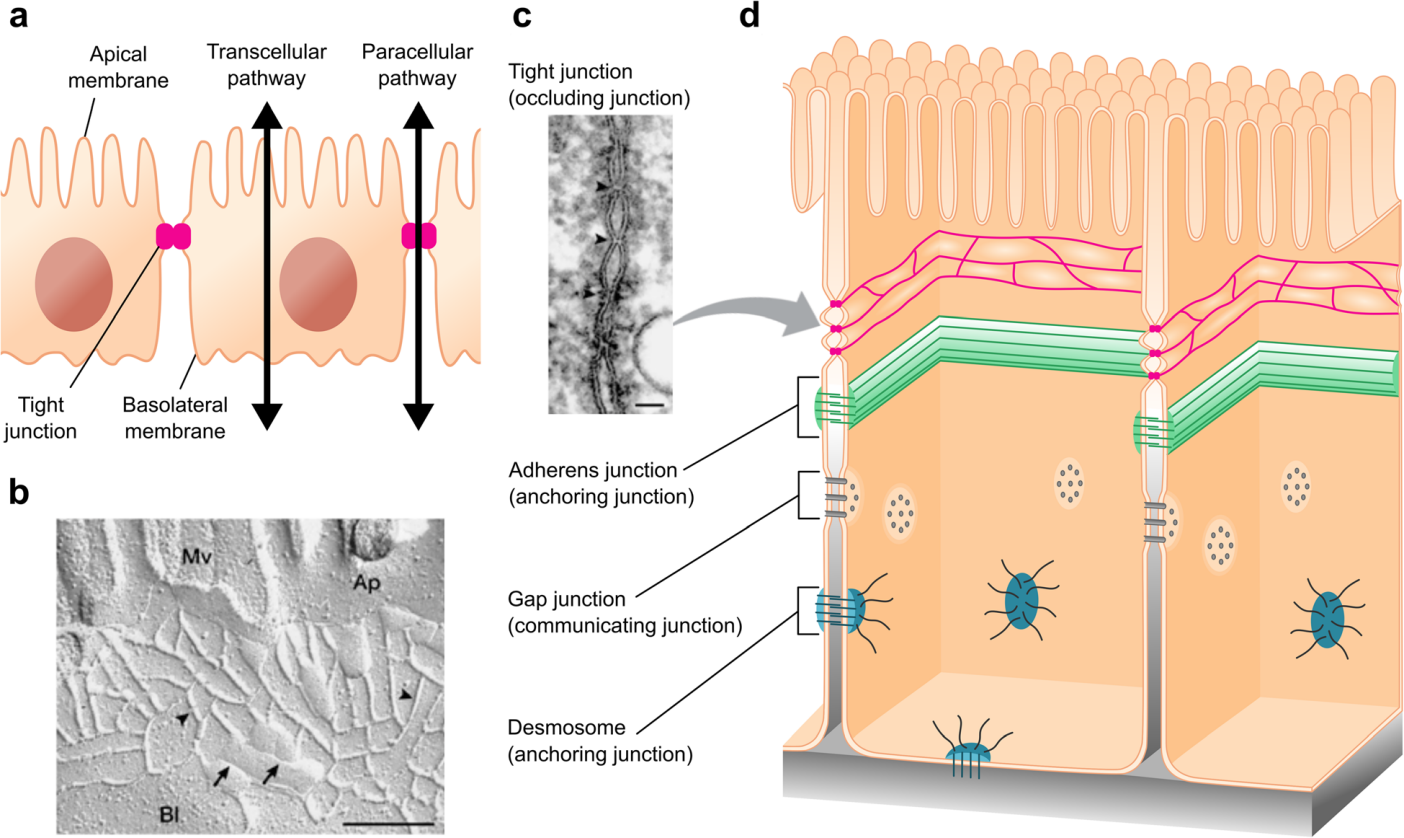
Transepithelial transport routes and intercellular junctions. **a** Paracellular and transcellular pathways across an epithelial layer. **b, c** Structure and localization of TJ strands including (**b**) freeze-fracture replica electron microscopic image (scale bar, 200 nm) and (**c**) ultrathin sectional view (scale bar, 50 nm). Mv, microvilli; Ap, apical membrane; Bl, basolateral membrane. Adapted by permission from Macmillan Publishers Ltd.: Nature Reviews Molecular Cell Biology [1], copyright 2001. **d** Schematic representation of the intercellular space and the junctional protein complex

Epithelial and endothelial sheets are formed by cells that are attached together sealing the intercellular space and thus providing a physical barrier. This tissue configuration leads to two possible routes for solutes to cross the barrier: 1) the transcellular pathway in which ions and molecules pass through the cell membrane and 2) the paracellular pathway where solutes cross between cells (Fig. 1a). Ion and molecule movement along the paracellular route is passive and requires a driving force such as a concentration gradient, an electrical potential difference, a hydrostatic pressure, or an osmotic gradient. The combination of both chemical gradient and electric voltage leads to an electrochemical gradient which accounts for most of the ion passive transport. Moreover, to move substances against their electrochemical gradient, cells have an active transcellular mechanism of transport (i.e., active transport) involving transmembrane proteins. This active transport coexists with the passive one in the transcellular route.

The permeability of cellular barriers is very dynamic and responds to extracellular stimuli and mediators by a cascade of signalling mechanisms [15] involving a cross-talk between paracellular and transcellular pathways [16]. For example, the kidney finely controls the whole body balance of calcium, phosphate, and magnesium by regulating the reabsorption of these ions in the renal tubule [17]. Another signalling cascade is initiated in blood vessels resulting in increased permeability when they are subjected to inflammatory stimuli, such as thrombin or tumour necrosis factor alpha (TNF-α) [18].

### Barrier function

The proper functioning of a barrier tissue depends on the polarization of cells by different membrane lipids and protein on their apical and basolateral sides [19]. The close contact between cells makes possible this apicobasal membrane polarity preventing the intermixing of specialized apical and basal membrane composition. Each cell type has its particular junctional complex with a mixture of different types of cell-cell junctions (Fig. 1b–d). There are basically four types of well-differentiated intercellular junctions between adjacent cells: 1) gap junctions channels link the cytoplasm of two cells and serve to exchange ions and small molecules, 2) desmosomes and 3) adherens junctions contribute to physically anchor the cells together, and 4) tight junctions (TJ) bring closer apposed membranes, being primarily responsible for controlling the passive transport of solutes through the paracellular pathway [20, 21].

TJ comprise a complex protein network [22], including transmembrane proteins found within plasma membrane as a branching network of strands. Each strand in turn is associated with another strand from the apposed membrane creating close contact points, the so-called ‘kissing points’, in which the intercellular space is obliterated (Fig. 1c). The number of strands is variable and differs among cell types. Claude et al. observed that the junctional tightness was logarithmically related to the number of strands instead of proportionally related, which led him to propose the existence of permeable pores that dynamically and randomly open and close for short periods of time [23]. New high-resolution approaches to detect flux across individual transmembrane protein channel suggest the possible existence of the dynamic behaviour, proposed many years ago by Claude et al., in the sub-millisecond timescale [24, 25]. Thus, TJ could finely regulate the barrier permeability by this dynamic gating in addition to the more well-known modulation by protein remodelling. In addition to the barrier function, TJ also act as a fence that restricts the diffusion of protein complexes and lipids within the plasma membrane. Thus, the TJ define the interface between apical and basal domains.

The understanding of TJ has vastly increased in the recent years and particular junctional proteins have been linked to specific barrier functions. However, the underlying mechanisms regulating such functions remain to be determined [3]. It is now clear that TJ not only physically seal the intercellular space but also form permeable channels that have charge and size selectivity [26, 27]. This selectivity supports the wide variety of permeability properties among epithelial and endothelial tissues, which is critical to maintain and regulate the fluid composition between distinct compartments in the organs. Therefore, their deregulation involves functional disorders in vital organs.

In the brain, diverse neurological diseases—stroke, epilepsy, Alzheimer’s disease, and multiple sclerosis, among others—share common blood-brain barrier (BBB) disruption that contributes to the severity of such diseases [28]. The BBB is formed by brain capillaries that separates the blood from the central nervous system (CNS) [29, 30]. In the retina, alterations of the blood-retinal barrier (BRB) [31]—which is similar to the BBB but protecting the nervous tissue of the retina—are directly associated with two leading cause of blindness in developed countries: diabetic retinopathy and age-related macular degeneration. Brain capillary endothelium is among the tighter endothelia in the human body, in which well-developed TJ severely restrict the passage of solutes. Unlike continuous capillaries such as the ones in the BBB and BRB, sinusoids are discontinuous and fenestrated (i.e., cell perforated) capillaries with high permeability, which facilitate the diffusion of solutes. The unique liver sinusoidal endothelial cell (LSEC) phenotype—including fenestration and lack of a basement membrane—is vital in maintaining functional hepatocytes. Dysregulation of the LSEC phenotype is an early stage of liver fibrosis that can progress to cirrhosis and liver failure [32].

Systemic inflammation can disrupt biological barriers; for instance, a persistent inflammatory state in the gut can compromise brain barriers and contribute to neurodegenerative disorders via the gut-brain axis [33, 34], which permits the dialogue between the gastrointestinal system and the CNS. Intestinal microbiota regulates CNS activity [35] and can promote these inflammatory conditions that in turn impair the intestinal barrier (i.e., epithelium and mucosa) and contributes to pathogenesis of inflammatory bowel diseases, such as Crohn’s disease and ulcerative colitis [36]. In the kidney, tubular epithelial cells that form the renal tubule may play a key role in driving interstitial inflammation and fibrosis that progress to chronic kidney disease (CKD) [37]; after an acute kidney injury, renal epithelium damage may be an early stage of renal failure. Furthermore, acute lung injury can damage the integrity of the alveolar-capillary barrier (which separates air from blood at the alveoli in the lung) resulting in a fluid leakage from capillaries to alveolar spaces and a subsequent pulmonary oedema with a potential risk of respiratory failure [38].

### Cell microenvironment

**Fig. 2:**
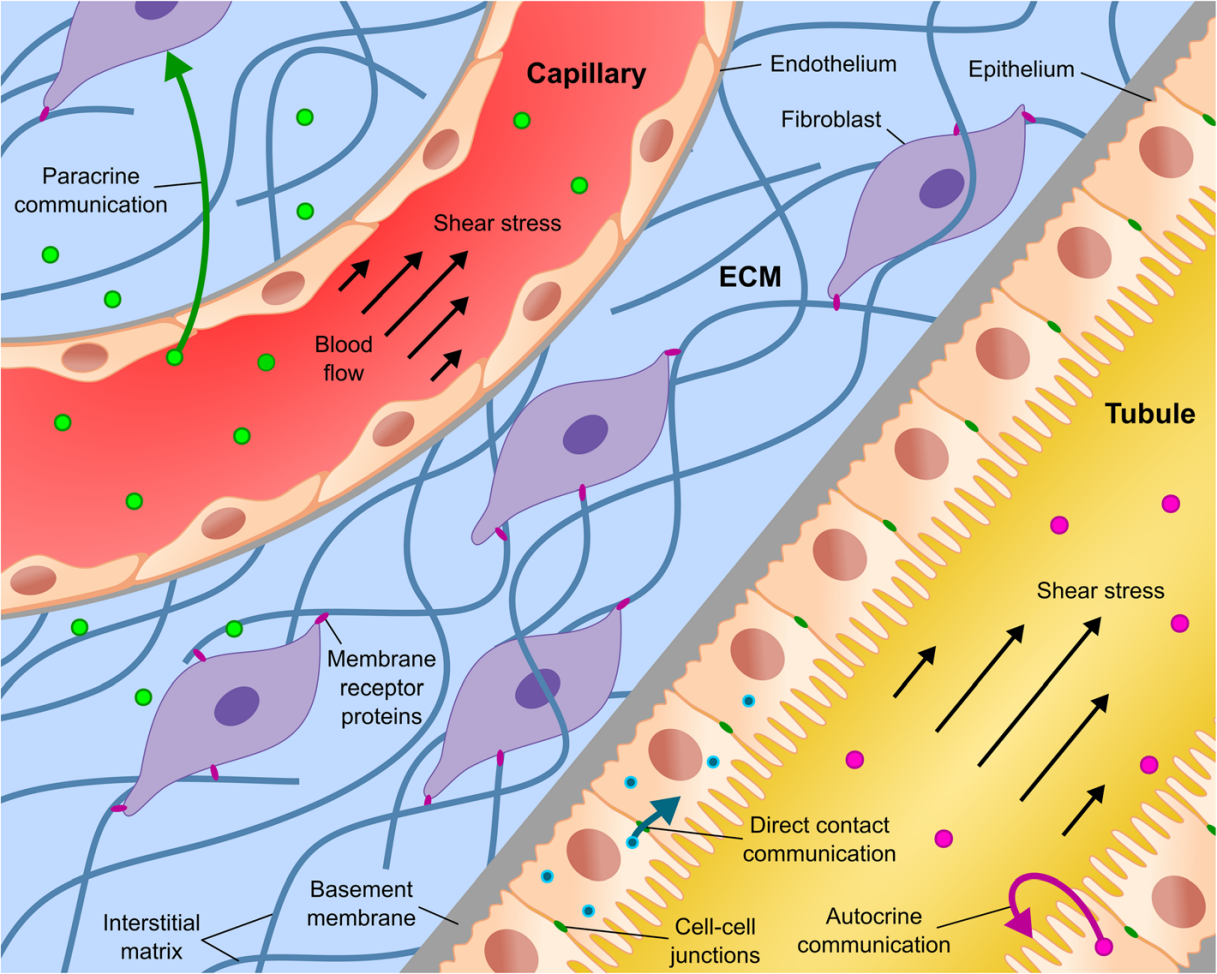
Physical and biochemical cues in the cell microenvironment. Schematic drawing of an epithelial tubule and an endothelial capillary embedded within an ECM. It includes cell-cell communications (i.e., direct contact, autocrine, and paracrine communications), flow-induced shear stress, ECM involving basement membrane and interstitial matrix components, and cell-ECM interaction through membrane receptor proteins

Many microenvironment factors critically influence epithelial and endothelial tissues (Fig. 2). For example, most epithelia produce an extracellular matrix (ECM) that support them and interface with other tissues acting as a diffusion barrier for the exchange of molecules. Moreover, cells react to physical and biochemical stimuli in the surrounding environment, which allows them to interact with other cell types and to dynamically adapt their barrier function to particular physiological needs.

#### Extracellular matrix (ECM)

The ECM is a three-dimensional (3D) structure fundamentally composed of water, proteins, and polysaccharides that physically supports cells within the organs [39]. Components of the ECM are produced by cells and secreted into the extracellular space where they are assembled into diverse structures, primarily collagen fibres. There are two general organizations of ECM depending on their localization and composition: 1) the basement membrane, which is a sheet-like layer mostly composed of collagen IV and laminins that separates epithelia from connective tissue; and 2) the interstitial matrix, which surrounds cells forming a porous scaffold for tissues and mainly consists of collagen I and fibronectin. The ECM regulates many essential cellular functions such as proliferation, migration, differentiation, and cell fate [40, 41]. Rather than being a rigid and static matrix complex, the ECM is constantly interacting with surrounding cells and remodelling itself to maintain tissue homeostasis. Cell-ECM interaction is mediated via biochemical signals coming from membrane receptor proteins (e.g., integrins) that link the matrix to the cytoskeleton and activate intracellular signalling pathways. Thus, ECM dictates important cellular behaviours. For example, Cell-ECM adhesion by specific membrane receptors marks an asymmetry of the cell surface that is involved in the establishment of cell polarity [42].

#### Cell-cell communication

Cell to cell interaction through messaging signals is fundamental to cooperate and thus succeed in the maintenance of tissue homeostasis. This communication is typically established using signalling biomolecules and can be divided into four categories: 1) direct contact in which cells share some binding molecules or in which small molecules diffuse between apposed cells via gap junctions, 2) autocrine signalling where a molecular messenger is released and subsequently bound to the same cell, 3) paracrine signalling where the signal reaches a nearby cell, and 4) endocrine signalling in which hormones travel long distances through the bloodstream and reach distant target cells. Heterotypic cell-cell interaction is necessary to maintain the barrier function of most epithelial and endothelial tissues. For example, although the BBB is physically created at the vascular endothelium, surrounding cells (e.g., pericytes, astrocytes, and neurons) play an important role in the barrier regulation [43]. Pericytes are essential during BBB development [44], and their lack or the disruption of their interaction with endothelial cells disrupt the BBB integrity [45, 46].

#### Biochemical cues

Some soluble factors such as hormones, cytokines, and growth factors serve as extracellular molecules for cell signalling and regulate fundamental cellular processes in barrier tissues. For example, growth factors stimulate cell growth, cell proliferation, and cell differentiation. In addition, a multitude of cytokines modulate epithelial barrier functions. In the gut, mucosal inflammation increases epithelial permeability via TJ disassembly, in part because activated immune cells release certain inflammatory cytokines [47, 48]. Indeed, specific cytokine inhibitors restore homeostasis in intestinal inflammation. Similar increased permeability were demonstrated in other epithelia such as airway epithelium when these are exposed to certain cytokines such as TNF-α and interferon gamma (IFN-γ) [49]. In the BBB, interleukins (IL-1α, IL-1β, and IL-6) and TNF-α cause the disruption of the barrier increasing the ion permeability of the BBB [50, 51]. In contrast, other chemical agents like some hormones (e.g., adrenomedullin and hydrocortisone) have the opposite effect on the BBB by tightening and strengthening the barrier [52, 53].

#### Physical cues

Cells are also able to sense the external physical environment such as the topography and the stiffness of the ECM. These physical features are transduced into a biochemical response through integrin-based adhesions complexes in a process known as mechanotransduction [54]. In particular, matrix stiffness regulates proliferation, migration, and cell-cell adhesion, among others, and its stiffening can lead to the progression of tumour angiogenesis [55], while the topography of the underlying ECM influences cell morphology and cell differentiation, among others [56].

Other physical forces, like the hemodynamic shear stress and intercellular forces through adherent junctions, coexist in the cell microenvironment and critically influence cell function and cell behaviour. Endothelial cells lining blood vessels sense hemodynamic shear stress acting on vessel walls. A sustained physiological variation of the shear stress has been found to stimulate vascular remodelling and to increase nitric oxide production by endothelial cells [57, 58]. In addition, many in vitro and in vivo studies have reported a shear-dependent endothelial permeability [59]. As in blood vasculature, shear stress is also a relevant physiological stimulus for epithelial tissues. For example, renal tubular epithelial cells are exposed to a shear stress on account of the filtrate flowing across the tubular lumen and are able to transduce this mechanical force at microvilli and primary cilia processes.

## Barrier function quantification

The ability to quantify the transepithelial transport in a barrier model is essential. Most common laboratory techniques are based on permeability assays and transepithelial electrical measurements. The former can be used to determine apparent permeability coefficients of test compounds (e.g., to predict drug absorption), whereas the later provides information on transepithelial ion transport (e.g., to assess barrier integrity). Since TJ have charge and size selectivity, the permeability quantification of charged and uncharged species often gives complementary information [60].

### Permeability assays

Permeability assays consist in tracer diffusion measurements in which the tracers are added in a donor compartment (i.e., the apical or basolateral side) and quantified in a received compartment (i.e., the opposite side) along time. Tracers can be monitored using a fluorescent dye (e.g., fluorescein isothiocyanate [FITC] and Rhodamine) or a radiolabelled marker (e.g., [^14^C] mannitol). In a single capillary, the apparent permeability coefficient (in cm s^− 1^) can be calculated considering a lineal relation between fluorescent intensity and the amount of the compound as

1 $$ {P}_{\mathrm{app}}=\frac{1}{\varDelta {I}_{\mathrm{f}}}{\left(\frac{dI_{\mathrm{f}}}{dt}\right)}_0\frac{r}{2} $$

where *I*_f_ is the fluorescent intensity in an optical window containing the capillary, ∆*I*_f_ is the fluorescence intensity increase after perfusing the lumen with a fluorescent labelled tracer, (*dI*_f_/*dt*)_0_ is the initial rate of increase in fluorescent intensity, and *r* is the capillary radius in cm [61]. Alternatively, in cell cultures, the *P*_app_ for a compound is calculated as

2 $$ {P}_{\mathrm{app}}=\frac{dQ}{dt}\frac{1}{A{C}_0} $$

where *dQ/dt* is the steady-state flux in μmol s^− 1^, *A* is the cell culture area in cm^− 1^, and *C*_0_ is the initial concentration in the donor compartment in μmol. This methodology permits to assess the transepithelial transport in both directions, distinguishing between active and passive transport mechanisms. For example, if the *P*_app_ calculated in the apical to basolateral direction is higher than in the opposite direction, it involves an active uptake of such compound. Additionally, specific transporter inhibitors can elucidate their transport contributions. To ensure the barrier integrity during permeation assays, especially at the end of the experiments, these studies are usually combined with transepithelial electrical measurements or also with transport studies using hydrophilic paracellular markers (e.g., mannitol and dextran). Detailed information on the protocol of permeability assays can be found in ref. [62].

Most common applications of permeability assays aim to predict drug permeability across cellular barriers. In the pharmaceutical industry, permeability assays using Caco-2 monolayers are the gold standard to predict intestinal absorption and thus to estimate human drug oral absorption [63]. In addition, assays using tracers with different molecular weights were crucial to reveal the size-selectivity of the TJ and to estimate their porosity and pore size [64, 65].

### Transepithelial electrical measurements

Transepithelial electrical measurements permits to quantify ion permeability and barrier function in cell cultures. Its major advantages are the non-invasiveness and the real timing of the measurement. On the contrary, tracer assays may be more sensitive, especially in leakier cell monolayers, but require long time periods for tracers to diffuse and need labelling. As a disadvantage, these measurements are difficult to interpret because they are the result of a combination of many cellular electrical parameters. For this reason, some assumptions and simplifications are usually considered to interpret the results. This technique is based on the measurement of the electrical properties of the cellular sheet by means of extracellular electrodes. In particular, a voltage or current perturbation is applied between electrodes placed in both sides of the barrier. The measurements that give primary information about a cellular barrier are transepithelial voltage (*V*_te_), transepithelial electrical resistance (TEER), and short-circuit current (*I*_sc_). These parameters are related by Ohms law as

3 $$ \mathrm{TEER}=\frac{\varDelta {V}_{\mathrm{te}}}{\varDelta I} $$

where ∆*V*_te_ is the change in *V*_te_ and ∆*I* is the change in the current flowed.

#### Transepithelial voltage

In epithelial tissues, the transepithelial ion transport is driven by active transporters, chemical gradients, and electrical forces due to potential differences. The net movement of ions across epithelia may lead to a charge imbalance between apical and basolateral sides that generates a transepithelial potential difference; this will be the sum of both apical and basolateral membrane potential differences.

#### Transepithelial electrical resistance

TEER is the paracellular sum of two ion conductive pathways, the paracellular resistance and the transcellular resistance. This parameter allows to monitor the cell barrier integrity during experiments and to electrically characterize barrier tissues. Epithelia and endothelia are traditionally grouped in two classes according to their transepithelial resistances: 1) ‘leaky’ epithelia when they have low TEER values and lower paracellular resistance compared to the transcellular one, and 2) ‘tight’ epithelia in which the TEER is much higher and the paracellular and transcellular resistances are similar. The BBB is an example of tight barrier with high selectivity and low permeability, which allows to protect the brain from potentially harmful substances (e.g., bacterial toxins and infectious agents); in situ measurement of TEER is reported to be 1462 Ω cm^2^ in rats [66] and 1850 Ω cm^2^ in frogs [67]. In the kidney, the distal nephron also presents a tight barrier in order to preserve the diluted filtrate coming from previous tubular segments (850 Ω cm^2^ in the distal tubule and 1000 Ω cm^2^ in the collecting duct [68]). Otherwise, the proximal tubule of the nephron is a leaky barrier that permits rapid paracellular transport of ions and water molecules from the lumen to the interstitial space (6–10 Ω cm^2^ [68]).

#### Short-circuit current

Under particular experimental conditions in which there is no electrochemical gradient across the epithelium (i.e., apical and basal solutions are the same, and *V*_te_ is clamped to zero), active ion transport generates a measurable electrical current, the so-called ‘short-circuit current’ (*I*_sc_). Although this current represents the net transport of all ions species, *I*_sc_ variations when removing such ion from the solution could estimate the active transport of one ion species.

#### Equivalent electric circuit and impedance analysis

**Fig. 3:**
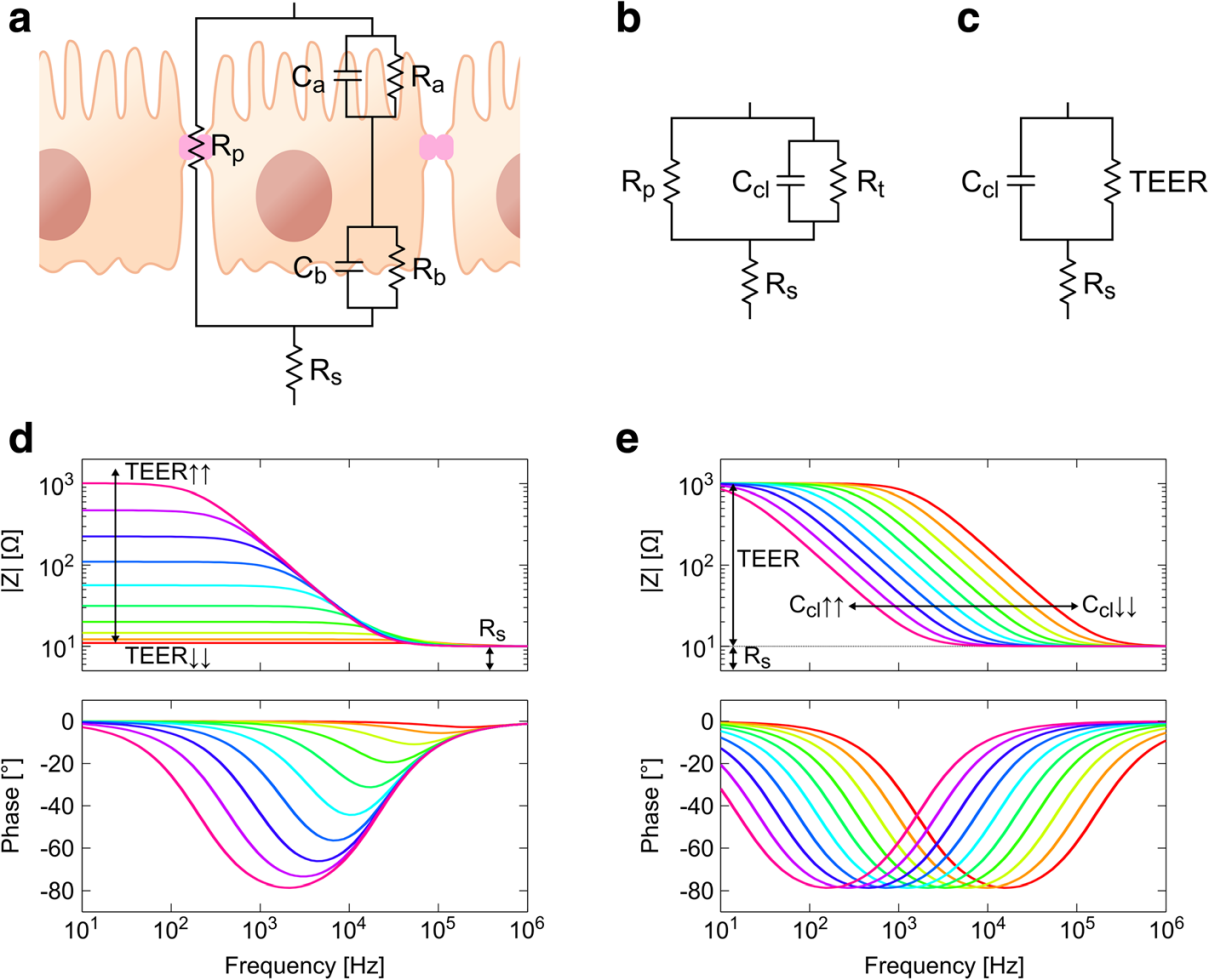
**a** Equivalent electric circuit for an epithelial cellular layer. **b** Model with lumped apical and basolateral elements. **c** Lumped model including only transepithelial electric resistance (TEER) and cell layer capacitance (C_cl_). **d**–**e** Representative impedance spectra across a cell layer; data was simulated with R_s_ equal to 1 kΩ, **d** TEER values ranging 1–1000 Ω, and **e** C_cl_ values ranging 0.1–10 μF. R_p_, paracellular resistance; R_s_, resistance of the solution; R_a_, apical resistance; R_b_, basolateral resistance; C_a_, apical capacitance; C_b_, basolateral capacitance; R_t_, transcellular resistance

The transepithelial impedance across a cellular monolayer can be described in terms of an electric circuit containing resistances and capacitances: 1) two capacitances (C_a_ and C_b_) that represent the apical and the basolateral cell membranes, 2) two resistances (R_a_ and R_b_) that describe the ion permeability of the cell membranes, and 3) a paracellular resistance (R_p_) to represent the selective ion route through the TJ (Fig. 3a). An additional resistance (R_s_) is often added in the circuit to describe the solution resistance between the electrodes and the cell layer. This five-variable model has to be solved with additional measurements; however, it is possible to obtain a two-variable model by lumping the elements. In a first step, the contributions from apical and basolateral membranes are grouped in a single transcellular resistance (R_t_) and a cell layer capacitance (C_cl_) (Fig. 3b). This can be done if R_a_C_a_ and R_b_C_b_ are similar. In a second step, the transcellular and the paracellular resistances are grouped in the TEER (Fig. 3c). Representative impedance spectra obtained across a cell layer are shown in Fig. 3d and e for the frequency range of interest (~ 10 Hz–1 MHz). An advantage of impedance spectroscopy is the discrimination between R_s_ and TEER unlike measurements systems using a single frequency.

Although the simplified model containing the TEER and the C_cl_ provides primary information about the cell barrier, sometimes it is necessary to solve the entire system. For example, it is often interesting to determine the particular ion permeability of TJ or to distinguish the electrical parameters of both membranes. For such purposes, one of the five parameters can be independently obtained and used to determine the others. In other approaches some parameters may be neglected when they do not contribute to the overall impedance (e.g., the TEER is dominated by the R_p_ in leaky epithelia where the R_p_ is much lower than the R_t_).

### Measurement techniques

#### Ussing chamber

**Fig. 4:**
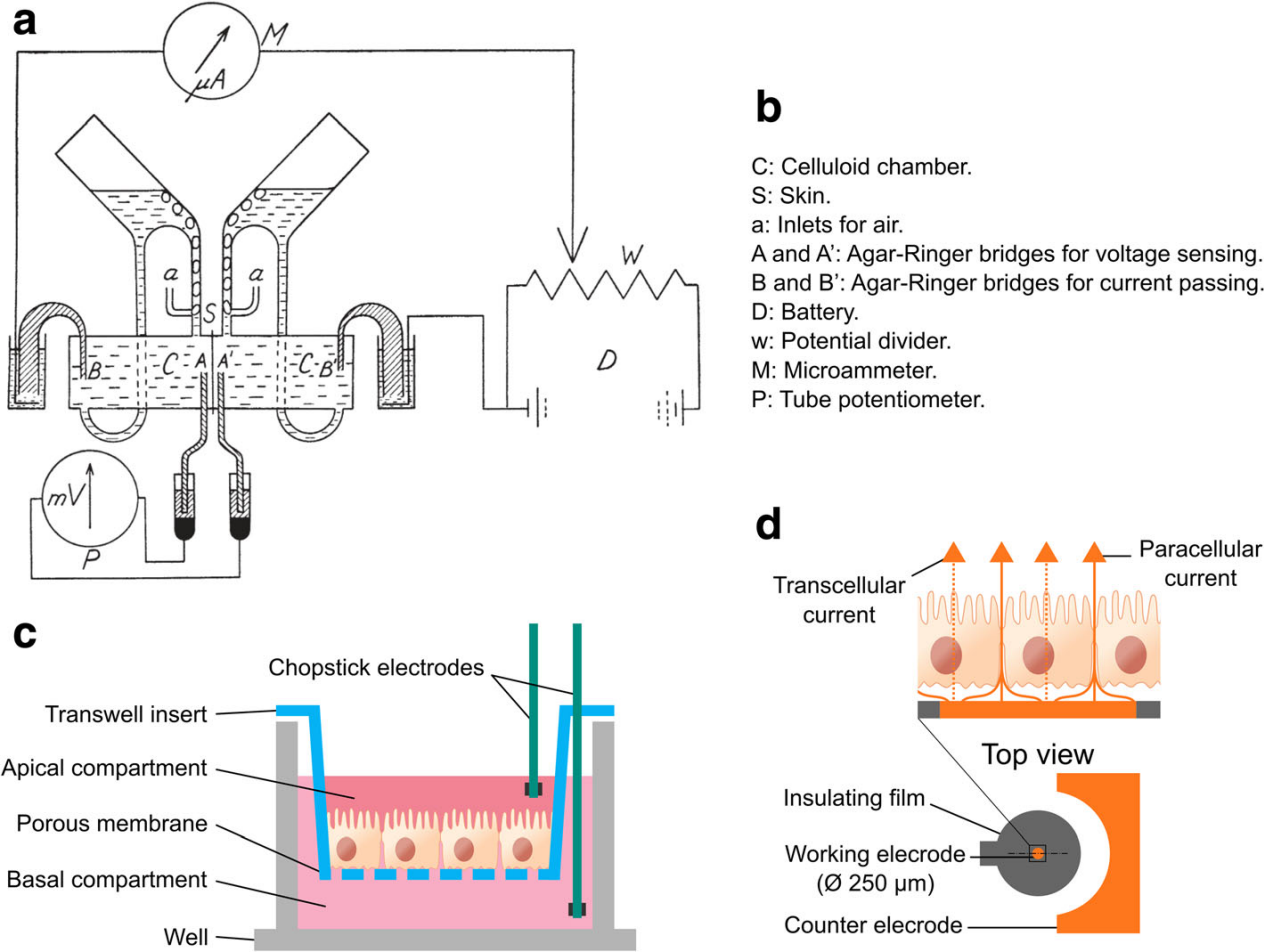
Transepithelial electrical measurement techniques. **a** Original diagram representation of the Ussing chamber in 1951. Reproduced by permissions of John Wiley and Sons [70]. **b** Detailed parts of the Ussing chamber. **c** Schematic representation of chopstick-like electrodes for use with standard Transwell inserts. **d** Electric Cell Substrate Impedance Sensing system. In this technology, cells are cultured on a surface that contains a small gold electrode (working electrode) and a large counter electrode

The Ussing chamber was an apparatus developed by the Danish biologist Hans H. Ussing [69] in the early 1950s to measure the active transport of sodium in frog skin epithelium. Ussing and Zerahn demonstrated that the rate of active transport of ions can be calculated as an electric current across the skin if both sides are at the same potential and have similar solutions [70]. By imposing these conditions, there would be no net transfer of passive ions, and the electric current (*I*_sc_) would result only from active transport processes. The original diagram representation of the apparatus is shown in Fig. 4a and b. It is composed of two compartments containing the same Ringer’s solution (appropriately oxygenated through the air inlets) and the piece of tissue under study separating them in the middle. To measure the potential difference and to pass the current across the tissue, four agar-Ringer bridges link the Ringer’s solution in the compartments with reference electrodes and current carrying electrodes, respectively.

Transepithelial measurements that can be obtained from Ussing chamber include *V*_te_, TEER, and *I*_sc_. By combining transepithelial measurements with chemically determined net fluxes, Ussing was able to prove that the overall *I*_sc_ measured across the frog skin was produced by the active transport of Na^+^ [70]. This was followed by other works where he demonstrated that the active transport of Na^+^ in reality is a forced exchange of Na^+^ against K^+^ located at the inward-facing membrane [71] and also revealed the passive transport origin of the Cl^−^ ions [72], both remain unchanged today. Other authors rapidly adopted the Ussing chamber to study other tissues (e.g., intestinal [73], urinary bladder [74], and kidney [75]).

To date, our knowledge about active and passive transport mechanisms in epithelial tissues has vastly increased thanks to the Ussing chamber; the versatility of the apparatus is unquestionable. A primary advantage of this system is the high accuracy of the measurement because of the uniform current distribution along the epithelial sheet. Commercially available Ussing chambers maintain the main features of the original device. A common Ussing-like chamber can accommodate a variety of tissues using interchangeable sliders even to hold a Snapwell insert (cell culture insert supported by a detachable ring).

#### Transwells

Transwell permeable supports are cell culture inserts to be used with standard well plates mainly for transport and migration studies. In general, these devices simply comprise an insert body with a bottom filter plate. The membrane filter serves as a cell growth substrate and permits particles to diffuse through the filter pores. Transwell supports have been very popular since the 1950s [76] due to their simplicity and their commercial availability. Unlike cells on culture dishes, cells grown on permeable supports have a basal side in which cells can uptake and secrete molecules. This is essential to provide an environment that better mimics the in vivo scenario and in turn enables cell polarization. Moreover, Transwell system facilitates the collection of samples from both tissue sides therefore permits transport studies. Some of the human cell barriers modelled with Transwell systems are the alveolar epithelium [77], BBB [78, 79], and intestinal mucosa barrier [80, 81].

The electrophysiological quantification of the barrier in Transwell cultures can be done by using a commercially available handheld probe based on chopstick-like electrodes (Fig. 4c). The probe comprises a pair of sticks with electrodes at the ends. Each stick in turn has a current carrying electrode (outer Ag electrode pair) and a pick-up electrode (inner Ag/AgCl pair). With this tetrapolar (four electrodes) configuration, it is possible to measure either the TEER or the *V*_te_. The procedure to determine the TEER requires an additional measure for quantifying the contributions of the medium solution to the total resistance. This is done by measuring the resistance with the Transwell insert free of cells (blank reading). Then, TEER can be computed as

4 $$ \mathrm{TEER}=\left({R}_{total}-{R}_{blank}\right)A, $$

where *A* is the area of the cell culture (i.e., the effective surface area of the Transwell insert). In order to compare TEER values, the TEER needs to be normalized by the effective area so that it is expressed in units of Ω cm^2^.

A disadvantage of chopstick-like systems is the proper positioning of the sticks and the non-uniform current distribution when large membrane filters are used (> 12 mm in diameter) [82]; in such cases, the resistance reading should not be converted to unit area resistance. To overcome these limitations, chopstick electrodes can be replaced by the so-called ‘Endohm series‘ (World Precision Instruments, Sarasota, FL, US). This system comprises a chamber and a cap each containing a pair of concentric electrodes. Therefore, the applied current is more uniformly distributed through the whole area providing reproducible and accurate measurements.

#### ECIS

Electric cell-substrate impedance sensing (ECIS) is a technology in which cells are cultured on a surface that contains sensing electrodes (Fig. 4d) [83]. The most basic ECIS cultureware for monitoring barrier function includes a small circular gold electrode of 250 μm in diameter surrounded by a relative large electrode. Since the working (also known as sensing) electrode is much smaller than the counter electrode, the volume around the working electrode contributes more to the measured impedance than volumes far away. Cells grow on top of this small electrode obstruct the current path that crosses the barrier, increasing the electrical resistance. Thus, the impedance measurement directly reflects the tightness of the cell barrier [84]. Monitoring of the barrier tightness in real time has been useful in determining the role of specific proteins in the regulation of endothelial barrier function and the barrier restoration from an induced inflammatory stimulus [85]. By using different electrode designs, apart from the barrier function, ECIS technology can be used to monitor cell attachment and spreading [86], cell migration, cell proliferation, cell invasion, and cytotoxicity [87]. Electrodes can be also used for wound healing assays apart from monitoring. For such wounding purpose, a temporally elevated voltage is applied to the electrode causing the death of the cells therein. Then, the healing process can be followed in real time [88].

The number of cells that covers an ECIS electrode of 250 μm in diameter is generally in the order of hundreds; otherwise the diameter of the smallest commercially available insert is around 4.2 mm in diameter which allocates 3 orders of magnitude more cells. As a result, measurements performed with ECIS technology are more sensitive to cell motility and shape of small populations of cells than methods that evaluate larger cell cultures or tissues. Another benefit of such experiments is the throughput and the reproducibility because of the fixed electrodes. As a disadvantage, measurements with ECIS electrodes are only representative of small areas. In addition, transport studies are not allowed in ECIS cultureware since there is no basal accessible compartment. To create this basal region, ECIS instrumentation can be combined with insert-like filter supports by means of an adapter (e.g., 8 W TransFilter Adapter, Applied Biophysics Inc., Troy, NY, US).

## Engineered biological barrier models

**Fig. 5:**
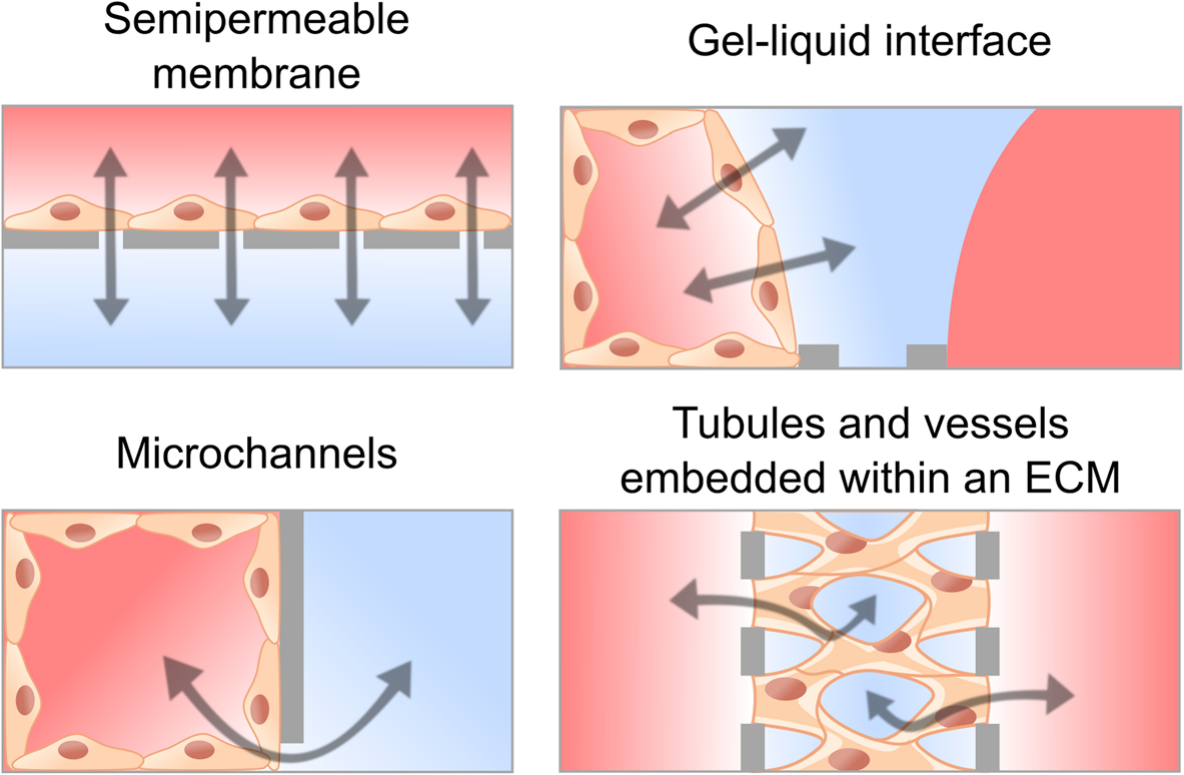
Compartmentalized approaches for engineering cellular barriers including membrane-based microfluidic device, side-by-side compartments connected through microchannels, gel-liquid interface using phaseguides, and perfusable tubules and microvascular network within an ECM

The early strategy to separate apical and basolateral domains of cell monolayers was by means of permeable supports. Epithelial cells on these membrane filters form polarized monolayers with transport and permeability qualities of in vivo transporting epithelia [89, 90]. Beyond these traditional cultures, MPS are refining in vitro models by partially emulating the cell microenvironment and the microarchitecture of native tissues. Recent advances in biomaterials and microfabrication technologies have allowed scientists to attempt novel compartmentalization approaches to develop barrier models. In the following, we discuss the applicability of the methods for the quantification of barrier permeability in those models and describe existing strategies for modelling biological barriers involving microengineering technologies, such as bioprinting, which comprises those 3D printing techniques capable of depositing biological elements (e.g., living cells and biomaterials); photolithography from the microelectronics industry; and soft lithography, which allow processing more physiologically relevant and low cost materials (e.g., elastomeric polymers). Current compartmentalized approaches are shown in Fig. 5.

### Membrane-based devices

This approach is based on a semipermeable membrane, where the cells are cultured, that split a chamber into two compartments. Most of these devices are made of thermoplastic or elastomeric materials such as polydimethylsiloxane (PDMS), which is transparent and biocompatible. Permeability assays in these devices are often easy to perform since solutions can be collected from both apical and basolateral compartments. However, it presents a major challenge to achieve the uniform current distribution required for accurate TEER measurements within miniaturized cell culture channels of MPS. Several researchers, including us, have proposed particular strategies and electrode configurations to address this issue and to allow monitoring of the barrier state in real time [82, 91–94].

A device based on this strategy is commercially available by Emulate Inc. (Boston, MA, US) with a main field of applications in drug development and toxicity. In a recent study, it was successfully used to retrospectively predict the prothrombotic side effect of a drug candidate for treatment of autoimmune disorders that was unknown during preclinical testing and was only revealed in human clinical trials [95]. Similar membrane-based systems have been widely used in the literature to model the BBB [96, 97], the alveolar-capillary barrier [8, 98], the hepatic sinusoid [99, 100], and renal tubular epithelia [101, 102], among others. For example, Blundell et al. constructed an in vitro model of placental barrier, which protects the foetus from harmful substances and regulates the transport of nutrients from the maternal to the foetal blood [103]. For this purpose, a co-culture of human trophoblasts and foetal endothelial cells were plated on either side of the semipermeable membrane under flow conditions. The model reproduced the efflux transport of a gestational diabetes drug (i.e., glyburide), mostly by active transporters in trophoblast cells [104]. In vitro models that faithfully recapitulate transport functions of placental barrier are essential to predict the exposure of foetus to drug compounds that may compromise foetal development during pregnancy.

**Fig. 6:**
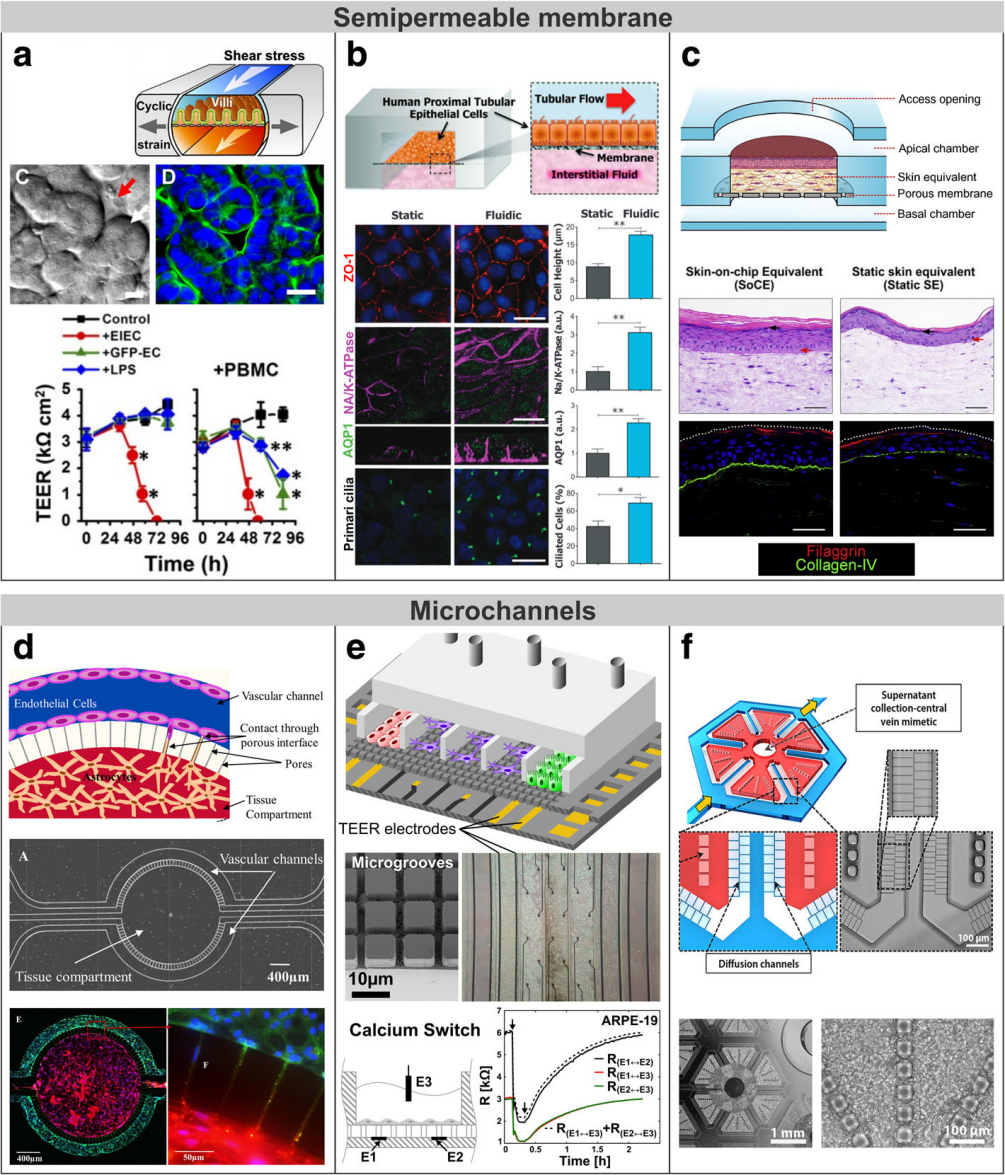
Engineered biological barrier models involving semipermeable membranes or microchannels. **a** A gut-on-a-chip microfluidic device with spontaneously formation of villi resulting from mechanical cues [107]. TEER profile shows intestinal barrier injury in the presence of pathogenic bacteria (EIEP) or immune cells plus either non-pathogenic bacteria (GFP-EC) or lipopolysaccharide (LPS) endotoxin. **b** A human kidney proximal tubule-on-a-chip. Immunofluorescence images and bar plots shows increased cell height and increased expression of the tight junction protein ZO-1, aquaporin 1 (AQP1; green), Na/K-ATPase (magenta), and primary cilia in epithelial cells under flow conditions. Adapted from [109] with permission from The Royal Society of Chemistry. **c** Microfluidic platform for the development of human skin equivalents. Histological and immunofluorescence images demonstrate an improved epidermal morphogenesis and dermoepidermal junction when the tissue is maintained in a dynamic air-liquid interface. Adapted from [110] with permission from Elsevier. **d** Neonatal BBB model consisted of side-by-side chambers connected through microchannels [114]. Immunofluorescence image shows direct contact communication between endothelial cells (ZO-1; green) and astrocytes (astrocytic marker GFAP; red). **e** Microfluidic model of the BRB where cells are arranged in parallel compartments and interconnected through a grid of microgrooves [10] – Adapted by permission of The Royal Society of Chemistry. TEER measurement during a calcium switch procedure is performed with two electrodes in the basal side instead of in the apical and basal sides. **f** Scalable liver-on-a-chip microdevice for long-term maintenance of hepatocyte function in vitro, in which microchannels artificially mimic the fenestrated endothelial cells of the liver [115] (Copyright IOP Publishing. Reproduced with permission. All rights reserved)

Instead of using a rigid permeable support, a flexible membrane (e.g., PDMS) can be utilized to apply a mechanical stretching to the cells. With this approach Hug et al. created a ‘lung-on-a-chip‘ that models the alveolar-capillary barrier [8]. They co-cultured microvascular endothelial cells in a compartment containing culture medium together with alveolar epithelial cells in a superimposed compartment filled with air, obtaining a tighter barrier (i.e., higher TEER) in this air-liquid interface culture condition than when the tissue was submerged in liquid. Since epithelium and endothelium are in the two sides of the semipermeable membrane, it is not possible to determine the TEER value of each cell monolayer. The authors managed to cyclically stretch the tissue using a vacuum system at the sides of the compartments thus mimicking the breathing motion of the lung. This system was able to reproduce the effects of a pulmonary oedema (including detrimental of the pulmonary barrier measured through FITC-inulin transport) by administrating a cytokine (interleukin-2) in the microvascular side concurrent with the cyclic mechanical strain [105]. The same device was also used to create a human intestinal model using Caco-2 cells. In order to increase the absorptive surface area, enterocytes—the specialized absorptive cells of the intestinal epithelium—form finger-like processes that project into the lumen (villi). Interestingly, the application of mechanical cues—fluid flow and mechanical deformation as in gut peristalsis—spontaneously undergoes the formation of villi and crypts, including a higher glucose uptake rate compared to cells cultured in a Transwell insert and differentiation of Caco-2 cells into four different cell types [106]. In another study, it was analysed whether mechanical deformations of villi affects bacterial overgrowth and inflammation [107]. The study demonstrated that cessation of epithelial deformation triggers bacterial overgrowth, and also that immune cells and endotoxin induce villus injury and intestinal barrier breakdown (as indicated by the decrease of TEER) because of the production of proinflammatory cytokines by epithelial cells (Fig. 6a).

Mechanical forces due to fluid flow in blood vessels and epithelial tubules can be reproduced in microfluidic devices to mimic an in vivo-like environment. For instance, Jang et al. created a device with a microchannel lined by kidney tubular cells from the collecting duct [101]. Results demonstrated that cells subjected to fluidic flow exhibit enhanced cell polarization, cytoskeletal reorganization, and molecular transport in comparison with cells grown on glass substrate under static conditions. In addition, the device was used to investigate the role of fluid shear stress in translocation of aquaporin-2 (a vasopressin-regulated apical water channel) [108]. A model of kidney proximal tubule was also developed in that microfluidic device for nephrotoxicity assessment [109], where proximal epithelial cells were subjected to flow and exposed to the nephrotoxin cisplatin (Fig. 6b). The tubule-on-a-chip better predicted nephrotoxicity in the human kidney compared to conventional cultures and even reproduced nephrotoxicity recovery after injection of cimetidine which has shown to suppress its toxic effects in canines. A dynamic environment was also used to promote the development of human skin equivalents. Sriram et al. cultured keratinocytes on a fibrin-based dermal matrix containing fibroblasts (i.e., a dermal equivalent) previously integrated into a microfluidic platform for continuous perfusion and ventilation of the tissue (Fig. 6c) [110]. By maintaining the tissue in the dynamic air-liquid interface, the skin model exhibited improved epidermal differentiation, barrier function (i.e., lower permeability), and a more robust dermoepidermal junction compared to skin equivalents cultured in standard tissue culture inserts. Importantly, the dermoepidermal junction had a basement membrane with increased expression of proteins from the collagen and laminin families, which are essential for cells to attach to the ECM and thus prevent epidermal detachment.

Membrane-based microfluidic devices have also been designed to allocate several epithelial and endothelial cell monolayers. Wufuer et al. fabricated a device composed of three layers of PDMS together with two intermediate porous membranes. They developed a ‘skin-on-a-chip‘model that mimics the cell layer physiology of the human skin consisting of epidermal, dermal, and endothelial layers [111]. The authors optimized a model of skin inflammation and oedema by applying TNF-α, as revealed from the delocalization of the TJ protein ZO-1, increased permeability to FITC-dextran (4 kDa), and an elevated pro-inflammatory cytokine profile. In addition, they also demonstrated the ability of the device for drug testing by reversing the pathological scenario through the administration of dexamethasone to treat the induced inflammation. A cell sheet stratification technique has also been used to construct several layered tissues. This technique is based on harvesting and stratifying contiguous cell sheets on a culture insert by means of a custom-made manipulator. Since the tissue lies on an insert membrane, it is possible to conduct TEER measurement and permeability assays as in Transwell systems. Using this methodology, Kim et al. fabricated a stacking of three cell layers mimicking the hepatic plate (i.e., hepatocytes forming layers of 1–2 cell thick) in the liver surrounded by two monolayers of sinusoidal endothelial cells [112]. Similar 3D cell stacking can be currently constructed by using bioprinting techniques. Horvath et al. [98] created alveolar-capillary barriers in vitro by a sequence of prints, including an endothelial cell layer, an ECM layer, and an epithelial cell layer. The methodology demonstrated high reproducibility and capability to achieve a very thin tissue barrier, comparable to the physiological thickness of the basement membrane found in native tissues (1.6 μm) which is critical for an adequate oxygen uptake. Artificial materials (e.g., plastic semipermeable membranes) used as a cell support can partially restrict the diffusion of solutes or a direct contact communication between heterotypic cells.

### Microchannels

Microchannels that physically link two side-by-side chambers have also been used as a compartmentalization strategy [113]. In this approach, cells are cultured in one of the chambers to form a cell monolayer that completely covers the inner surfaces. With a microchannel size of a few microns, epithelial or endothelial cells lining the chamber would be unable to pass through the microchannels whereas biomolecules could diffuse through them. In addition, supporting cells in the adjacent compartment could extend cytoplasmic processes to enable a direct contact between heterotypic cells (e.g., astrocytic end-feet extended along the microchannels). The planar disposition of the compartments enables real-time fluorescence imaging of the whole cell culture to calculate the *P*_app_ of test compounds. In this kind of systems, *V/S* substitutes *r*/2 in Eq. 1 where *V* is the volume of the vascular compartment and *S* is the permeable surface (i.e., the area of the microchannels). Otherwise, TEER measurements can be conducted by integrating electrodes in both compartments or by threading wire electrodes through the outlets and inlets. However, special care has to be taken in expressing the TEER in units of Ω cm^2^ since the current distribution may be unobvious in these systems with microchannels.

A microfluidic chip based on microchannels is commercially available from SynVivo Inc. (Huntsville, AL, US). The device consists of a PDMS slab containing the fluidic microstructures bonded to a glass slide, which facilitates real-time imaging. In a work by Deosarkar et al., the SynVivo chip was used to develop a model of BBB by culturing rat brain capillary endothelial cells with either astrocytes or an astrocyte conditioned medium (Fig. 6d) [114]. Both cell cultures exhibited improved barrier formation that was supported by permeability to tracers and electrical resistant measurements, in contrast to just endothelial cultures. Interestingly, *P*_app_ of 40 kDa dextran closely mirrored the permeability of rat brain capillaries in vivo.

In our group, we developed a compartmentalized microfluidic device to allow the real-time monitoring of several cell monolayers and their heterotypic cell-cell interaction (Fig. 6e) [10]. The device included crisscross microgrooves on a glass substrate to address the challenge of interconnecting several tissue-tissue interfaces. In addition, electrodes were integrated in the substrate for the electrophysiological monitoring of several barrier tissues. As a proof-of-concept, we used it to recapitulate the cell structure of the retina mimicking both inner and outer blood-retinal barriers.

Engineered microchannels have also been used as a microfluidic endothelial-like barrier. Several authors have artificially reproduced the very leaky endothelium of the liver sinusoid by the microchannels themselves (Fig. 6f). Despite the lack of cell functionalities (i.e., no endothelial cells), a non-biological barrier can simulate some mass transport properties of blood vessels to maintain for long term the phenotype and functions of primary hepatocytes in cell cultures [115, 116].

### Gel-liquid interface

**Fig. 7:**
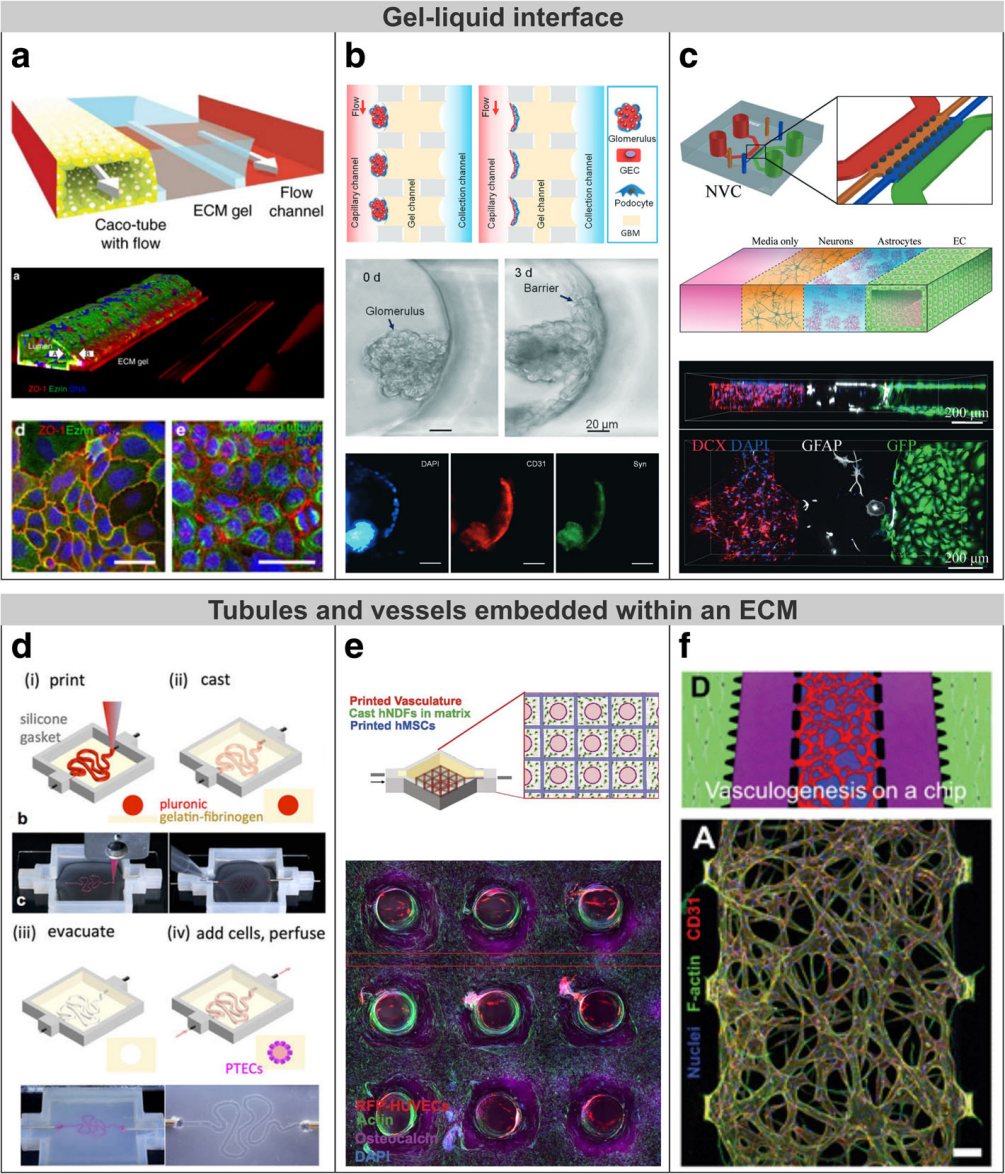
Engineered biological barrier models involving gel-liquid interface or tubules and vessels embedded within an ECM. **a** Intestinal epithelium tubule in a microfluidic channel created with a gel interface [118]. As revealed by immunofluorescence, cells form a confluent layer lining the whole channel which results in a perfusable lumen. **b** Glomerulus-on-a-chip microdevice. Glomerular microtissues are adhered to a gel surface and self-assembled into a continuous barrier of endothelial cells (CD31; red) and podocytes (synaptopodin; green) under flow perfusion. Adapted from [120] with permission from The Royal Society of Chemistry. **c** Three-dimensional neurovascular microfluidic model that enables heterotypic cell-cell interactions; it comprises human microvascular endothelial cells mimicking cerebral blood vessels, primary rat neurons, and astrocytes. Adapted from [121] with permission from The Royal Society of Chemistry. **d** Bioprinting method for creating 3D human renal proximal tubules in vitro that are fully embedded within an ECM, including printing of a sacrificial ink, casting of an ECM, evacuation of the ink, and cell seeding [9]. **e** Three-dimensional bioprinting of thick vascularized tissue consisted of a perfusable vascular network using hUVECs surrounded by hMSCs and fibroblasts [129]. Immunofluorescence image shows osteogenic differentiation (Osteocalcin; violet) of hMSCs in situ after administration of specific growth factors via the vascular network. **f** A perfusable microvascular network grown in a hydrogel channel. The obtained network exhibits relevant morphological characteristics of in vivo blood vessels. Adapted from [134] with permission from The Royal Society of Chemistry

Gel-liquid interfaces can physically support the cells while enabling a direct contact interaction between tissues. These interfaces can be microfabricated by means of phaseguides structures that act as capillary pressure barriers [117]. The function of these structures is to selectively pattern a gel in a central lane by meniscus pinning, so cells could proliferate in the interface between the gel (e.g., an ECM hydrogel) and the liquid (e.g., culture medium) in adjacent lanes. This technology is commercially available by Mimetas (Leiden, NL) in a high-throughput microfluidic platform called OrganPlate. Trietsch et al. used it to develop up to 40 replicas in a single platform of a model of intestinal tract epithelium (Fig. 7a) [118]. They created perfusable polarized epithelial tubes using Caco-2 cells and assessed its barrier integrity during drug-induced cell death. For that purpose, fluorescent tracers are added in the luminal side and time-lapse fluorescence images are automatically taken in the ECM hydrogel. Then, the *P*_app_ of a tracer may be calculated as in microchannels approach using Eq. 1; or also effective concentrations and exposure times of drug compounds may be determined for pharmacological studies. Unlike cells cultured on 2D monolayers, it is difficult to carry out transepithelial electrical measurements on perfusable tubules where cells cover all the walls of a channel. Therefore, in vitro models of tubules or vessels often only combine immunocytochemistry and permeability assays to quantify the transepithelial transport.

A hydrogel channel can also be defined using equally spaced post structures. The surface tension caused by these structures will prevent the hydrogel from entering in between the posts [119]. Wang et al. developed a microfluidic device able to trap primary glomerular microtissues into ECM semilunar substrates and succeed in forming glomerular filtration barriers including endothelial cells, basement membrane, and podocytes that entirely covered the ECM surface (Fig. 7b) [120]. In particular, the microdevice was found suitable for modelling diabetic nephropathy—a chronic loss of kidney function in people with diabetes mellitus—since microtissues under high glucose conditions exhibited, in a dose-dependent manner, barrier dysfunction, increased albumin permeability, and excessive reactive oxygen species production, which are closely associated in the progression of diabetic nephropathy. To permit co-cultures and thus emulate heterotypic cell-cell interactions, cells can be added to the gel preparation before filling the channels. For instance, a neurovascular unit consisting of human microvascular endothelial cells mimicking cerebral blood vessels, primary rat neurons, and astrocytes was constructed with multiple side-by-side ECM and fluidic channels (Fig. 7c) [121]. Moreover, gel-liquid approach has enabled cell migration studies in response to specific cytokines such as tumour cell intravasation (i.e., migration towards blood vessels) under TNFα exposure [122] or tumour cell extravasation (i.e., migration from blood vessels) under chemokine CXCL5 [123]. In another work, cell migration caused by epithelial-mesenchymal transition (EMT) (a biological process by which polarized epithelial cell acquires a mesenchymal phenotype) was initiated in proximal tubular epithelial cells covering a basement membrane extract in a microfluidic device [124]. In response to serum proteins, cells become myofibroblasts and migrated within the gel. EMT has been proposed to be an early stage of fibrotic disorders, such as kidney interstitial fibrosis which is characterized by an excessive accumulation of ECM in the interstitial space. Since observation of EMT process is extremely difficult in vivo, MPS have become relevant in vitro tools to reproduce and follow in real-time this process.

Three-dimensional characteristic features of native tissues have been reproduced in in vitro by structuring gel surfaces. For example, hydrogel scaffolds with an array of finger-like elements that mimic in vivo villi were developed to support intestinal epithelial cells [125]. For this purpose, a microfabricated alginate mould was used to create the 3D scaffold by pouring a pre-gel solution that was subsequently polymerized and detached from the mould after dissolving the alginate [126]. In that study, the expression of MUC17 (which is a transmembrane mucin responsible for the protective layer of mucus against pathogens) was higher in presence of the 3D organization than in 2D models and, interestingly, its knockdown compromised the intestinal barrier function. On the other hand, TEER measurements and tracer assays have evidenced a leakier barrier for intestinal epithelial cells cultured on 3D villi-like structures compared to Transwell inserts [125, 127], which is accounted for a decreased expression of tight junctions [128] and an increased cell culture area in the 3D model.

### Tubules and vessels embedded within an ECM

The latter microengineered approach consists in embedded tubules or microvascular networks into an ECM. Such epithelia and endothelia can be constructed either by creating fluidic microchannels that are later lined with cells or by forming channels of hydrogel laden with cells that results in branching microvessels. The primary advances of these approaches are the relevant 3D architecture and the capacity to perfuse the microvessels or tubules. In this approach, there are no straightforward solutions to determine the electrophysiological properties of 3D architectures (e.g., microvessels with circular cross-sectional geometries) contained within microchannels; the access to microvessels embedded in microchannels is limited unlike isolated tubules that can be cannulated or in situ measurements where vessels can be micropunctured. For such purpose, permeability assays with fluorescent markers offer an accurate way to check the integrity of microvessel networks within microchannels.

Homan et al. reported a bioprinting methodology to create 3D renal proximal tubules on demand (Fig. 7d) [9]. They constructed a convoluted tubule by printing a sacrificial ink that was embedded within an ECM and subsequently removed. Finally, the convoluted tubular channel (~350 μm in diameter) was filled with cells, perfused with culture medium, and allowed to form a monolayer lining the channel. The 3D in vitro tubule besides recreating a cyclosporine-dependent nephrotoxicity exhibited enhanced epithelial phenotype (e.g., cell height, microvilli development, and albumin uptake) than conventional 2D cell cultures.

Similar bioprinting methodology was employed by Kolesky et al. to develop a perfusable vascular network contained by an ECM (Fig. 7e) [129]. They managed to obtain a thick (> 1 cm) 3D fluidic network interconnecting various lattice patterns of sacrificial ink. After liquefying and evacuating the ink, channels of the network were lined with human umbilical vein endothelial cells (HUVECs) to form a vascularization system. In addition, hMSCs and fibroblasts were incorporated in the surrounding ECM as a parenchyma and connective tissue, respectively. The long-term administration of specific growth factors via the vascular network promoted the osteogenic differentiation of hMSCs in situ; thus being able to recapitulate heterogeneous complex tissue architectures and enabling its maturation.

Another methodology is the so-called ‘viscous finger patterning’ that can be used to create lumens with circular cross-sectional geometries. For this purpose, a droplet of culture medium is placed on the inlet of a microchannel previously loaded with pre-polymerized hydrogel solution. As a result, the culture medium flows through the centre of the microchannel displacing the hydrogel [130]. After polymerization of the hydrogel, there remains a patterned lumen that can be lined with endothelial cells to mimic a blood vessel [131]. Furthermore, endothelial cells can be combined with supporting cells by seeding them first on the luminal wall or by incorporating them in the hydrogel solution. For example, a co-culture of primary human brain microvascular endothelial cells, pericytes, and astrocytes was developed with this technique to mimic the BBB [132]. Under inflammatory stimulation with TNF-α and compared to Transwell cultures, the vascular system showed a profile of secreted cytokines that closely mimicked those observed in the living brain.

Relatively complex vascular structures can also be engineered using soft lithography techniques instead of 3D printing. For example, a scaffold made of biodegradable elastomer was constructed by stacking several microfluidic layers [133]. In that work, the authors built a scalable vascular system containing fluidic branches that were covered with hUVEC. Combining this engineered vessel network with parenchymal cells such as rat hepatocytes or cardiomyocytes, they constructed functional vascularized liver and cardiac tissues, respectively. In particular, the latter was implanted in rat femoral vessels via direct anastomosis, which supports its applicability for regenerative medicine.

Unlike systems with defined vasculature, the development of microvessel networks within an ECM can closely mirror their in vivo morphology. Kim et al. demonstrated the spontaneous formation of interconnected networks of microvessels in a microfluidic device using hUVEC (Fig. 7f) [134]. The device consisted of five side-by-side channels separated by posts, in which the central channel was filled with hydrogel. Either the hydrogel laden with the endothelial cells or the cells coating one of the sides of the hydrogel resulted in the proliferation of a long-term stable perfusable microvascular network; the sine qua non of this formation were growth factors secreted by supporting cells (i.e., human normal lung fibroblasts) that were co-cultured in a lateral channel. In addition, endothelial cells exposed to flow-induced shear stress exhibited some physiological responses (i.e., cytoskeleton reorganization and nitric oxide synthesis) of in vivo vessels. A very similar device was used to model choroidal neovascularization in age-related macular degeneration, wherein new blood vessels from the retinal choroid abnormally grew towards the retinal pigmented epithelium compromising the outer BRB [135]. They combined a perfusable blood vessel network embedded in an ECM with a cell monolayer of retinal epithelial cells attached to the ECM, where both tissues were separated by a gap channel. The administration of VEGF at high concentrations in the apical side of the retinal epithelium induced the proliferation of new vessels that penetrated the RPE monolayer. Otherwise, bevacizumab (a current drug for the age-related macular degeneration treatment) was found to inhibit choroidal neovascularization caused by VEGF and therefore vessel invasion. In a different approach, it was developed a platform based on a standard 96-well plate with six microfluidic units for similar purposes, including a hydrogel chamber for vasculogenesis and two microfluidic channels for continuous nutrients supply [136]. The device was found suitable for generating perfusable microvessels networks and especially for screening of drugs, as they tested the efficacy of well-known anti-angiogenic and anti-cancer drugs in a tumoral environment by co-culturing cancer cells.

## Conclusions and future perspectives

Epithelial and endothelial barriers are crucial to maintain organ homeostasis and their deregulation play an important role in the pathogenesis and progression of many prevalent human diseases. Most in vitro models of biological barriers, typically based on Petri dishes or Transwell inserts, are restricted to one or two different cell types and are inadequate to apply controlled physical or biochemical stimuli that emulate the in vivo microenvironment. Taking advantage of the available microfabrication techniques and biomaterials as well as the synergies between biologists and engineers, it has been possible to refine conventional in vitro models towards more sophisticated MPS able to better predict human response. Current MPS are heterotypic cell cultures with physiologically relevant organizations, where cells, for example, can be embedded in an ECM with a particular stiffness, seeded on a surface with certain topography, subjected to mechanical forces such as flow-induced shear stress and cyclic stretching, or exposed to concentration gradients of cytokines and growth factors.

In addition to facilitating real-time imaging, an advantage of many MPS modelling tissue barriers is the integration of electrodes for continuous monitoring of transepithelial electrical parameters. Nevertheless, obtaining accurate transepithelial measurements with these systems is not easy. To date, electrophysiology of the paracellular pathway where TJ reside has been addressed by measuring large cell culture areas with countless cells. It is likely that new tools emerge in the future able to record the ionic conductances of individual channels in tight junctions. This unprecedented spatial resolution measurement would give new insights into the underlying mechanisms of barrier regulation in molecular terms.

In vitro barrier models are suitable for studying the ability of pharmaceutical compounds to cross biological barriers, since these are the major impediment for agents to reach targeted tissues during drug delivery. In addition, barrier models are useful to quantify the transepithelial transport of drugs and thus provide information about times and doses at which organs are exposed to the drug for toxicity testing. Engineered MPS using human cells (e.g., primary cells or stem cells) have a high potential in many applications including drug screening, disease modelling, and regenerative medicine. In particular, the combination of these systems and stem cell technologies is a promising tool for the development of the so-called ‘precision medicine’, in which patient-derived cells are used for a personalized treatment.

Despite the progress made to develop representative MPS, many challenges remain to be addressed. For example, when dealing with heterotypic cell cultures, every tissue thrives in a specific cell culture medium that can differ from the others. Moreover, the heterogeneity of cells is detrimental for controlling the cell microenvironment of each cell type, which is a major advantage of MPS. On the other hand, microfabrication using soft lithography is appropriated to reproduce the cell organization of native tissues since it enables features in the sub-micron range; however, it requires the stacking of several patterned layers to build complex 3D structures, which results in a cumbersome fabrication process. Meanwhile, bioprinting technology is still in its infancy but is expected to grow rapidly if some issues related to print speed, bioinks viscosities, and printer cost can be improved. Another challenge is to find biomaterials compatible with the microengineering techniques and also inert to the bind of drugs and compounds. Finally, it is desirable to develop user-friendly MPS that could be handled for non-skilled personnel and adapted to common equipment in cell culture laboratories.

## Abbreviations

3D: Three-dimensional
BBB: Blood-brain barrier
BRB: Blood-retinal barrier
Caco-2: Colorectal adenocarcinoma cell line
CNS: Central nervous system
ECIS: Cell-substrate impedance sensing
ECM: Extracellular matrix
EMT: Epithelial-mesenchymal transition
hMSCs: Human mesenchymal stem cells
hUVEC: Human umbilical vein endothelial cells
LSEC: Liver sinusoidal endothelial cell
MPS: Microphysiological systems
PDMS: Polydimethylsiloxane
TJ: Tight junctions
TNF-α: Tumour necrosis factor alpha
VEGF: Vascular endothelial growth factor
